# Characterization of novel mycobacteriophages with a focus on phage Pisa4 and its interaction with human innate immune cells

**DOI:** 10.3389/fmicb.2026.1823245

**Published:** 2026-08-04

**Authors:** Andrea Bonacorsi, Alessandro Fusco, Davide Manessi, Fabio Filippini, Enrico Casu, Magda Marchetti, Ivana Palucci, Michela Sali, Michele Lai, Giulia Freer, Silvia Dominici, Giovanni Delogu, Federico Pratesi, Arianna Tavanti, Laura Rindi, Mariagrazia Di Luca

**Affiliations:** 1 Department of Biology, University of Pisa, Pisa, Italy; 2 Department of Translational Research and New Technologies in Medicine and Surgery, University of Pisa, Pisa, Italy; 3 National Centre of Artificial Intelligence and Innovative Technologies for Health, Istituto Superiore di Sanità, Rome, Italy; 4 Department of Basic, Clinical, Intensive Care and Perioperative Biotechnological Sciences – Section of Microbiology, Università Cattolica del Sacro Cuore, Rome, Italy; 5 Antimicrobial Resistance Laboratory, International Centre for Genetic Engineering and Biotechnology (ICGEB), Trieste, Italy

**Keywords:** antibiotic resistance, bacteriophage, innate immunity, intracellular bacteria, macrophages, mycobacteria, *Mycobacterium abscessus*, phage therapy

## Abstract

Four mycobacteriophages Pisa1, Pisa4, Florence1 and Florence4, taxonomically classified as new species, were isolated using *Mycobacterium smegmatis* mc^2^155 as a host strain. Genomic analysis revealed dsDNA genomes devoid of virulence or antibiotic-resistance genes. All encoded lysogeny-associated elements, but Pisa4 lacked the integrase gene and showed a partial immunity repressor gene, suggesting a potentially impaired lysogenic cycle. Phages exhibited siphoviral morphology, short replication cycles (≤30 min) and burst sizes of 50–150 PFUs/bacterial cell. All phages remained active between pH 4–12 and up to 45 °C. Given its potentially impaired lysogeny, Pisa4 was considered a promising candidate for antibacterial applications; therefore, its interaction with THP-1 macrophages was investigated. The phage was internalized in THP-1 and co-localized with its host both after co-incubation with bacteria prior to infection and upon post-infection administration. However, phage replication was restricted to the co-incubation condition. Importantly, Pisa4 did not trigger neutrophil extracellular trap release and elicited minimal cytokine responses in peripheral blood mononuclear cells, with no TNF-*α* or IFN-*γ* induction. In contrast, a significant increase in IL-6 production was observed at high phage concentrations, which may be influenced by co-purified bacterial components. Finally, Pisa1 and Pisa4 infected 12 and 8 out of the 48 *Mycobacterium abscessus* clinical isolates tested, respectively, while Florence phages were inactive. Collectively, these results highlight that Pisa phages, particularly Pisa4, are promising candidates for future therapeutic development.

## 1 Introduction

The rise of antibiotic-resistant bacteria is one of the most pressing global health challenges. Many antibiotics have lost efficacy and the pipeline for new antimicrobial agents is steadily contracting. This led to a growing number of infections that are increasingly difficult to treat with conventional antibiotics (World Health Organization, 2024: Bacterial Pathogens of Public Health Importance, to Guide Research, Development, and Strategies to Prevent and Control Antimicrobial Resistance, 2024). In this context, bacteriophages have re-emerged as promising alternatives or adjuncts to traditional antibiotic therapies. Phages are viruses that specifically infect bacterial cells, hijacking their machinery to replicate and ultimately causing host cell lysis (Strathdee et al., 2023). Due to their narrow host range, ability to self-replicate at the site of infection and potential to evolve alongside bacterial targets, phages are increasingly viewed as a viable strategy for tackling multidrug-resistant infections (Strathdee et al., 2023; Cesta et al., 2025). Mycobacteriophages (phages that infect the genus *Mycobacterium*) represent one of the most diverse and characterized groups of bacteriophages, largely due to extensive research initiatives such as the Phage Hunters program (Hatfull, 2018). Based on genomic similarity, they are classified into various clusters, with same cluster members sharing biological traits (Bonacorsi et al., 2024). Their genomes are typically mosaics, containing genes for the lytic cycle, with some also carrying genes for the lysogenic cycle. Among them, lytic phages are particularly attractive for therapeutic purposes (Hatfull, 2018). Although the majority of mycobacteriophages are temperate, often requiring genetic modifications to be safely used for therapy, a growing body of evidence supports their feasibility in treating drug-resistant mycobacterial infections. A milestone in the clinical use of mycobacteriophages was reported in 2019: a cocktail of three phages, two of which had been genetically engineered to convert them from temperate to strictly lytic, was administered to a cystic fibrosis (CF) patient suffering from a disseminated *Mycobacterium abscessus* infection. The therapy was well tolerated and achieved significant clinical benefits, representing the first documented therapeutic use of mycobacteriophages in a human patient (Dedrick et al., 2019). Later in 2023, Dedrick et al. (2023a) expanded upon this work with a description of a case series of twenty patients, including sixteen individuals with CF, who were treated with phage therapy for drug-resistant infections due to *M. abscessus*, *M. chelonae*, *M. bovis* and *M. avium*. This study, conducted under compassionate use regulations, contributed more evidence to the therapeutic value of phages, showing both safety and clinical efficacy. While the outcomes varied across patients, a substantial proportion showed favorable responses, reinforcing the need for further investigation and standardization of phage-based interventions. Although several mycobacteriophages have shown therapeutic potential, the number of phages that have been extensively characterized and evaluated for clinical applications remain limited. Furthermore, the genetic diversity observed among clinical mycobacterial isolates and the variability in phage susceptibility, often restrict the applicability of mycobacteriophages (Dedrick et al., 2019, 2023a). Therefore, the continuous isolation and characterization of novel mycobacteriophages is essential to expand the availability of therapeutic candidates, identifying phages with favorable features. Additionally, their clinical translation requires a deeper understanding of their interactions with the human immune system (Champagne-Jorgensen et al., 2023; Fajardo-Lubian and Venturini, 2023). In this study, we aimed to isolate and characterize novel mycobacteriophages for potential therapeutic use, in particular for targeting *M. abscessus*. Among them, we selected one phage that exhibited favorable features for potential clinical application and investigated its interactions with THP-1 macrophages using confocal microscopy. We also assessed the immunomodulatory effects of this phage, including neutrophil extracellular trap induction and cytokine production by primary human peripheral blood mononuclear cells. Finally, the lytic activity of the phages was also tested against a panel of *M. abscessus* strains, most of which were isolated from patients with CF.

## 2 Materials and methods

### 2.1 Bacterial strains and growth conditions

*M. smegmatis* mc^2^155 and *M. abscessus* ATCC 19977, obtained both by the American Type Culture Collection, as well as 47 *M. abscessus* clinical isolates (Table 1) and a strain of *M. smegmatis* mc^2^155 carrying the pMV231007 plasmid were used in this study. This plasmid carries genes codifying the DsRed protein and kanamycin resistance. All the strains were cultured in Middlebrook 7H9 (with or without 0.05% Tween80) or on 7H10 media supplemented with albumin dextrose saline (ADC) (10%) and glycerol (0.55%). Primary cultures were grown in 7H9 medium with Tween80, at 37 °C. These were diluted 1:100 in 7H9 medium without Tween80 to obtain secondary cultures, which were incubated on an orbital shaker (110 rpm) at 37 °C.

**Table 1:** *Mycobacterium abscessus* strains.

Strain ID	Subspecies	Morphotype	Source
2427	*M. abscessus*	R	Ospedale Cisanello, Pisa
2556	*M. abscessus*	R	Ospedale Cisanello, Pisa
2792	*M. abscessus*	S	Ospedale Cisanello, Pisa
2794	*M. abscessus*	S	Ospedale Cisanello, Pisa
2860S	*M. abscessus*	S	Ospedale Cisanello, Pisa
2860R	*M. abscessus*	R	Ospedale Cisanello, Pisa
2871	*M. abscessus abscessus*	S	Ospedale Cisanello, Pisa
2935	*M. abscessus*	S	Ospedale Cisanello, Pisa
2953S	*M. abscessus massiliense*	S	Ospedale Cisanello, Pisa
2953R	*M. abscessus massiliense*	R	Ospedale Cisanello, Pisa
2994	*M. abscessus abscessus*	S	Ospedale Cisanello, Pisa
3061	*M. abscessus massiliense*	S	Ospedale Cisanello, Pisa
3114	*M. abscessus bolletii*	S	Ospedale Cisanello, Pisa
3115	*M. abscessus abscessus*	R	Ospedale Cisanello, Pisa
3141	*M. abscessus abscessus*	S	Ospedale Cisanello, Pisa
3154	*M. abscessus abscessus*	R	Ospedale Cisanello, Pisa
3208S	*M. abscessus abscessus*	S	Ospedale Cisanello, Pisa
3208R	*M. abscessus abscessus*	R	Ospedale Cisanello, Pisa
3334S	*M. abscessus abscessus*	S	Ospedale Cisanello, Pisa
3334R	*M. abscessus abscessus*	R	Ospedale Cisanello, Pisa
3388	*M. abscessus abscessus*	S	Ospedale Cisanello, Pisa
R2S	*M. abscessus abscessus*	S	FPG, Roma
R2R	*M. abscessus bolletii*	R	FPG, Roma
R3S1	*M. abscessus bolletii*	S	FPG, Roma
R3S2	*M. abscessus abscessus*	S	FPG, Roma
R3S3	*M. abscessus abscessus*	S	FPG, Roma
R4S1	*M. abscessus abscessus*	S	FPG, Roma
R4S2	*M. abscessus abscessus*	S	FPG, Roma
R5S	*M. abscessus abscessus*	S	FPG, Roma
R5R	*M. abscessus abscessus*	R	FPG, Roma
R6S	*M. abscessus abscessus*	S	FPG, Roma
R6R	*M. abscessus abscessus*	R	FPG, Roma
R7S	*M. abscessus abscessus*	S	FPG, Roma
R7R	*M. abscessus abscessus*	R	FPG, Roma
R8S1	*M. abscessus massiliense*	S	FPG, Roma
R8S2	*M. abscessus massiliense*	S	FPG, Roma
R9S1	*M. abscessus abscessus*	S	FPG, Roma
R9R1	*M. abscessus abscessus*	R	FPG, Roma
R9S2	*M. abscessus abscessus*	S	FPG, Roma
R9R2	*M. abscessus abscessus*	R	FPG, Roma
R9S3	*M. abscessus abscessus*	S	FPG, Roma
R9S4	*M. abscessus abscessus*	S	FPG, Roma
MABS L	*M. abscessus abscessus*	S	FPG, Roma
MABS R	*M. abscessus abscessus*	R	FPG, Roma
AB S	*M. abscessus abscessus*	S	ISMETT, Palermo
AB R	*M. abscessus abscessus*	R	ISMETT, Palermo
MM	*M. abscessus abscessus*	R	ISMETT, Palermo

S: smooth; R: rough. FPG: Fondazione Policlinico Gemelli, ISMETT: Istituto Mediterraneo per i Trapianti e Terapie ad Alta Specializzazione.

### 2.2 Bacteriophage isolation, amplification and titration

The enrichment procedure was performed by using *M. smegmatis* mc^2^155 and *M. abscessus* ATCC 19977, along with five rough and five smooth clinical strains as hosts, according to Nayak and colleagues with some modifications (Nayak et al., 2023). Briefly, 5 g of soil samples were mixed with 10 mL of phage buffer (10 mM Tris–HCl (pH 7.5), 10 mM MgSO_4_, 68.5 mM NaCl, and 1 mM CaCl_2_) and incubated on a shaker for 3 h at room temperature (RT). The supernatant was collected and filtered (0.22 μm) after centrifugation at 5.200 x g for 30 min. The filtrate was mixed with 500 μL of a secondary culture of *M. smegmatis* in 7H9 medium without Tween80 twofold concentrated in a 1:1 ratio. The resulting culture was incubated overnight at 37 °C on an orbital shaker (110 rpm), centrifuged, filtered and spotted on a bacterial lawn. To prepare the lawn, 500 μL of the overnight *M. smegmatis* culture was mixed with 500 μL of ADC and 4 mL of 0.5% top agar containing 1 mM CaCl_2_, and the mixture was poured onto a 7H10 plate. The day after, plaques were individually picked using a sterile tip, transferred on a bacterial lawn and diluted using sterile paper strips. These plates were incubated at 37 °C overnight. This procedure was repeated until only one plaque morphology was obtained. Five mL of phage buffer were added to the plates, which were incubated overnight on a shaker at 4 °C. The day after, phage buffer was collected, centrifuged and filtered to obtain the phage stock. Tenfold dilutions of the phages were spotted on *M. smegmatis* bacterial lawn and, after 1–2 days of incubation at 37 °C, the phage titer was evaluated by plaque counting.

### 2.3 Phage genome extraction, sequencing and genotypic characterization

Phage genome extraction was performed following the Witold Kot protocol, with minor changes (Deng et al., 2019). Briefly, 180 μL of phage lysate were mixed with 1X DNAse I buffer and DNAse I (1 U/μL) (ThermoFisher Scientific). Following an incubation for 30 min at 37 °C, the enzyme was inactivated by adding EDTA (50 mM). Subsequently, SDS (1%) and Proteinase K (>600 U/mL) (ThermoFisher Scientific) were added, and the mixture was incubated for 45 min at 55 °C. The phage DNA was purified using the Zymo Research DNA Clean & Concentrator kit (Zymo Research) and quantified using Qubit 4 Fluorometer (Invitrogen). Illumina sequencing libraries were prepared by using the Illumina DNA Prep kit following manufacter’s instruction. Genomes were assembled using the Unicycler assembly tool (v0.4.8) and further analyzed using BLAST, Easyfig and RASTtk (Altschul, 1997; Sullivan et al., 2011; Olson et al., 2023; Altschul et al., 1990). Genome annotation was manually curated. To verify the absence of antibiotic resistance and virulence genes, the ABRIcate tool (version 1.0.1) was used employing different databases (Resfinder, CARD and VFDB) for cross-checking (Yukgehnaish et al., 2022). Taxonomic classification of the phages was performed according to the guidelines established by Turner and colleagues (Turner et al., 2021).

### 2.4 Transmission electron microscopy

Aliquots of 10 μL from each sample were placed onto formvar/carbon-coated 400-mesh copper grids (Agar Scientific, Essex, UK). Excess sample was removed and then 10 μL of 2% phosphotungstic acid (pH 7.0) was added to the grid for 30 s at RT and the excess was removed. Acquisitions were made using a FEI/Philips EM 208S transmission electron microscope (FEI, Eindhoven, Netherlands), operating at an accelerating voltage of 100 kV, equipped with acquisition system/Megaview SIS camera (Olympus, Hamburg, Germany).

### 2.5 pH and temperature stability assays

A modified version of the protocol from Sinha and colleagues was used for the pH stability assay (Sinha et al., 2020). Briefly, phage stability was assessed at different pH values (ranging from 3 to 12), using phage buffers adjusted with either NaOH or HCl. Phages (~1 × 10^9^ PFU/mL) were diluted 1:100 in the different phage buffers and incubated for 1 h at RT. For the temperature stability assay, aliquots of the phage suspension (~1 × 10^8^ PFU/mL) were incubated at different temperatures (ranging from 4 °C to 65 °C) for 1 h (Nayak et al., 2023). Following incubation, the samples were diluted and spotted on a lawn of *M. smegmatis* mc^2^155 for phage titer evaluation.

### 2.6 Phage life cycle evaluation

One-step growth curves were performed according to the following modified protocol (Catalão et al., 2011). Briefly, exponential phase bacteria (~3 × 10^7^ CFU/mL) were infected with phages at multiplicity of infection (MOI) of 1 and incubated for 50 min to allow phage particles to adsorb. Phages were then inactivated by adding 100 μL of H_2_SO_4_ 0.4% for 5 min. The H_2_SO_4_ was then neutralized with 100 μL of NaOH 0.4% and 150 μL of the culture were added to 15 mL of 7H9 without Tween80. This culture was incubated on an orbital shaker at 37 °C. Aliquots of 500 μL were collected every 30 min for the first 2 h, then every 2 h up to 6 and centrifuged at 16.000×*g* for 6 min. The supernatant was tenfold diluted and spotted for plaque counting.

### 2.7 Growth kinetic assay

A secondary culture of *M. smegmatis* mc^2^155 was diluted in 7H9 without Tween80 to reach an optical density (OD_600_) of 0.4 (~3 × 10^7^ CFU/mL) and further diluted to 10^6^ CFU/mL. Then, the phage was added at different MOIs (0.1, 1 and 10), alongside a growth control. The cultures were incubated at 37 °C in shaking conditions and sampled at different timepoints (0, 1, 3, 6, 24 and 48 h). Samples were diluted in a solution of phage inactivation buffer (PIB; 40 mM citric acid, 10 mM KCl, 135 mM NaCl, pH 3.0) to inactivate extracellular phages (Schmalstig et al., 2024), containing 0.05% Tween80, and plated on 7H10 medium for colony counting. Prior to the experiments, PIB treatment was validated to inactivate phage without affecting bacterial viability.

### 2.8 Phage purification in cesium chloride gradient

Phage lysates were purified by cesium chloride (CsCl) density gradient ultracentrifugation, as previously described (Fausti et al., 2025). Briefly, 0.5 g of CsCl (Sigma-Aldrich) were dissolved in 1 mL of phage lysate and the mixture was carefully layered onto a pre-formed, stepwise CsCl gradient with different density layers (ranging from 1.20 to 1.70 g/mL) prepared in ultracentrifuge tubes (Beckman Coulter). Samples were centrifuged at 150.000 × *g* for 24 h at 4 °C using a SW41 Ti swinging-bucket rotor (Beckman Coulter). After centrifugation, the phage opalescent band was collected using needle, transferred into dialysis tubes with a 10 kDa molecular weight cutoff (ThermoFisher) and dialyzed against 1.5 L of sterile phage buffer at 4 °C with gentle agitation. The buffer was replaced after 4 h, and the process was repeated overnight in fresh SM buffer. Following dialysis, the purified phage was recovered, filtered and titrated.

### 2.9 Confocal laser scanning microscopy (CLSM) of phage uptake and co-localization with its bacterial host within human macrophages

THP-1 cells were cultured in 75 cm^2^ flasks in RPMI 1640 (Gibco) supplemented with L-glutamine (Gibco), sodium pyruvate (Gibco) and 10% fetal bovine serum (FBS, Gibco), at 37 °C in 5% CO_2_.

For CLSM experiments, THP-1 cells were seeded in *μ*-slides 8 well (Ibidi) at 1 × 10^6^ cells/well and differentiated into macrophages by incubation with 0.01 μg/mL phorbol 12-myristate 13-acetate (PMA; Sigma) for 24 h at 37 °C in 5% CO₂, after which the medium was replaced with fresh RPMI 1640 to allow the monolayer to stabilize for an additional 24 h. Exponential phase cultures of *M. smegmatis* mc^2^155 expressing DsRed were washed twice with PBS and resuspended in half volume of RPMI 1640. The suspension was vortexed with 4 mm glass beads for 15 min to disrupt bacterial clumps and then left to sediment for 30 min to eliminate the remaining aggregates. The clarified suspension was diluted to achieve MOI of 1 (bacterium/human cell) for macrophages infection. Phages labelled with SYBR™ Gold Nucleic Acid Gel Stain (Thermofisher) were used in these experiments and administered using two modalities. In the co-incubation treatment, mixed phages and bacteria with an MOI of 10 (phage/bacterium) were immediately applied to the macrophage monolayers. The monolayers were then incubated for 3 h at 37 °C, after which cells were treated with RPMI 1640 containing 200 μg/mL amikacin for 1 h to eliminate extracellular bacteria prior to staining. In the post-infection treatment, instead, macrophages were first infected with bacteria for 3 h at 37 °C, the medium was replaced with RPMI 1640 containing 200 μg/mL amikacin for 1 h and, after extensive washing, purified phages at a concentration of 10^3^ phages/human cell were added to the monolayers for 3 hours before staining. Immediately before the acquisition, cell monolayers were stained using bisBenzimide H 33342 trihydrochloride (Sigma), for the nucleus, and CellMask™ Deep Red Plasma Membrane Stains (Thermofisher), for the membranes, and incubated at 37 °C for 15 min. Acquisitions were made using a Nikon AX/AX R confocal microscope (Nikon) equipped with a 63x oil immersion objective (1.4 numerical aperture) with an additional digital zoom factor of 3.5. Single section images were collected with a resolution of 2048 × 2048, the confocal pinhole was kept at 1, the gain and the offset were adjusted to prevent saturation of the brightest signal and minimal crosstalk scanning for each channel was performed. To evaluate colocalization, around 30 microscopy fields (around 40 cells per field) were captured and analyzed on Fiji software (Schindelin et al., 2012).

### 2.10 Determination of intracellular phage replication

THP-1 cells were seeded in 96-well plates at a density of 4 × 10^4^ cells per well. For these experiments, the MOI was set at 0.1. The same protocols used in the CLSM experiments were performed. Additionally, a third condition was introduced: in the pre-incubation treatment, the bacterial suspension was incubated with purified phages at an MOI of 10 for 50 min at 37 °C prior to addition to the macrophages. Notably, in the post-infection treatment, phage-containing supernatants were maintained throughout the experiment to promote continuous phage uptake by host cells over time. For phage quantification, at defined time points (0, 3, 24, and 72 h post-phage administration), cell media were collected. Macrophages were then washed three times with PBS and lysed in 0.1% Triton X-100. Cell media and lysates were centrifuged at 12.000 × *g* for 2 min. The resulting supernatants were serially diluted in PBS and spotted onto *M. smegmatis* lawns to determine extracellular and intracellular phage titers. In parallel, intracellular bacteria were quantified. In this case, culture medium was removed and monolayers were washed three times with PBS. Cells were then incubated in PIB containing 0.1% Triton X-100 to inactivate extracellular phages (Schmalstig et al., 2024) and subsequently lysed by pipetting. The lysates were serially diluted in PBS supplemented with 0.05% Tween80 and plated on 7H10 agar to enumerate intracellular bacteria.

### 2.11 Phage-induced neutrophil extracellular trap (NET) evaluation

Polymorphonuclear cells (PMN) were isolated from peripheral blood of healthy donors using a double density gradient centrifugation method as described by Petretto et al. (2019), with some adjustments. Briefly, 12 mL of blood were diluted to 20 mL with Dulbecco’s phosphate buffered saline (D-PBS) (Merk) and layered over a double Histopaque gradient (1.077/1.119) (Merk). The samples were centrifuged at 500 × *g* for 30 min. The granulocyte layer was collected and washed twice with cold D-PBS. Cells were diluted in Hank’s balanced salt solution (HBSS), 200 μL (1 × 10^5^ PMNs/well) were seeded onto sterile 13 mm round coverslips and incubated for 1 h (37 °C, 5% CO_2_). Neutrophils stimulation was performed as described by Roach and colleagues with some modifications (Roach et al., 2023). Briefly, cells were stimulated with purified phages (10^3^ PFUs/PMN) in HBSS with CaCl_2_ (2 mM) for 4 h. PMA (100 nM) was used as positive control and untreated cells as a negative control. Following incubation, PMN were fixed with paraformaldehyde (4% in PBS) for 10 min and blocked with normal goat serum (5%) and bovine serum albumin (1%) in PBS for 1 h, followed by incubation with rabbit polyclonal anti-myeloperoxidase antibody (Dako) diluted 1/300 in blocking buffer. After washing with PBS, cells were incubated with a goat anti-rabbit Alexa Fluor 488 secondary antibody (Thermofisher) (1:300) for 1 h in blocking buffer. Nuclei were stained with DAPI (Thermofisher) for 15 min. Slides were washed with PBS, mounted in glycerol and visualized using an Olympus BX-41 fluorescence microscope at 20x magnification. Five fields were analyzed per sample. Percentage of NETosis in each sample was obtained by counting double positive (DAPI/MPO) cells versus DAPI positive cells.

### 2.12 Phage-induced cytokine release by peripheral blood mononuclear cells

Peripheral blood mononuclear cells (PBMCs) were isolated from peripheral blood of healthy volunteers using density gradient centrifugation (Higdon et al., 2016). Following isolation, PBMCs were washed with D-PBS and resuspended in RPMI 1640 medium supplemented with 10% FBS. A total of 1 × 10^5^ cells/well were seeded into 96-well plates. The assay was designed based on previous studies, with some modifications (Van Belleghem et al., 2017; Perea et al., 2021; Popescu et al., 2022). Cells were incubated with medium only, phage buffer, *M. smegmatis* mc^2^155 and three different concentrations of purified phages (10^1^, 10^2^ and 10^3^ PFUs/cell) for 24 and 48 h at 37 °C with 5% CO₂. Cytokine production was measured in the supernatants of the cultured PBMCs using a DuoSet ELISA kit (R&D Systems, Bio-Techne, Minneapolis, MN, USA), according to the manufacturer’s instructions. The levels of interleukin-6 (IL-6), interferon-gamma (IFN-*γ*), and tumor necrosis factor-alpha (TNF-*α*) were determined. Cell culture supernatants were tested undiluted for IL-6 and IFN-*γ*, while TNF-α at a 1:3 dilution.

### 2.13 Host range and efficiency of plating (EOP) assay

The phage susceptibility of *M. abscessus* clinical isolates was tested by spotting tenfold dilutions of the phages (stock of 1 × 10^8^ PFU/mL) on bacterial lawns. The plates were incubated to evaluate the phage titer at 37 °C for 1–6 days. The efficiency of plating was calculated as:


$$\mathrm{EOP}=\frac{\frac{\mathrm{PFU}}{\mathrm{mL}}\mathrm{on}\;\text{tested strain}}{\frac{\mathrm{PFU}}{\mathrm{mL}}\mathrm{on}\;\text{host strain}}$$

EOP values equal to 0 mean a non-susceptible bacterium; values between 0 and 1 refer to a bacterium which is less susceptible than the host.

When only halos were visible, phages were tested in liquid bacterial cultures to determine whether they could replicate within the strains (Hyman and Abedon, 2010; Fausti et al., 2025). Briefly, secondary bacterial cultures were diluted 1:100 in fresh 7H9 medium without Tween80 and infected with phages at MOI 0.1 (phages:bacteria). Cultures were incubated for 24 h at 37 °C on an orbital shaker. The day after, cultures were centrifuged and the supernatant was diluted and spotted for plaque counting. The obtained titers were then compared to the initial one, with titer increases ≥ 0.5 log_10_ considered significant.

### 2.14 Statistical analyses and graphical representation

The statistical analyses were performed using Student’s *t* test, Multiple *t* test, Mann–Whitney U test and One-way ANOVA with Tukey post-hoc test, with a *p*-value threshold of <0.05. Graphical representations were done using GraphPad (version 9.5.1), depicting mean values with corresponding standard deviations.

## 3 Results

### 3.1 Isolation and genomic analysis of four novel mycobacteriophages

Four phages, named Pisa1 (GenBank Accession: PX093955), Pisa4 (GenBank Accession: PX093956), Florence1 (GenBank Accession: PX093957) and Florence4 (GenBank Accession: PX093958), were isolated from soil samples collected in Pisa or Florence (Italy), respectively, using *M. smegmatis* mc^2^155 as host. No phages were found using *M. abscessus* as host. All isolated phages had a dsDNA genome, and their characteristics are summarized in Table 2. BLASTn analyses revealed that Pisa phages were most closely related to *Mycobacterium* phage Urkel (GenBank Accession: KX657796.1), while Florence phages showed the highest similarity to *Mycobacterium* phage Bonamassa (GenBank Accession: OL455889.1) (Figure 1). According to the taxonomy guidelines established by Turner and colleagues (Turner et al., 2021), the newly isolated phages were classified as new species within their respective genera. Finally, Pisa phages belong to the genus *Anayavirus* and cluster K1, while Florence phages belong to the genus *Benedictvirus* and cluster A5.

**Table 2:** Genomic characteristics of phages Pisa1, Pisa4, Florence1 and Florence4.

Phage name	Genome length (kb)	GC%	Best match (% coverage–% Identity)	Genus	Cluster
Pisa1	60.44	66.65	Urkel 93%–93.25%	*Anayavirus*	K1
Pisa4	55.81	66.70	Urkel 94%–93.25%	*Anayavirus*	K1
Florence1	50.03	60.70	Bonamassa 94%–97.72%	*Benedictvirus*	A5
Florence4	49.09	60.75	Bonamassa 95%–97.72%	*Benedictvirus*	A5

**Figure 1:**
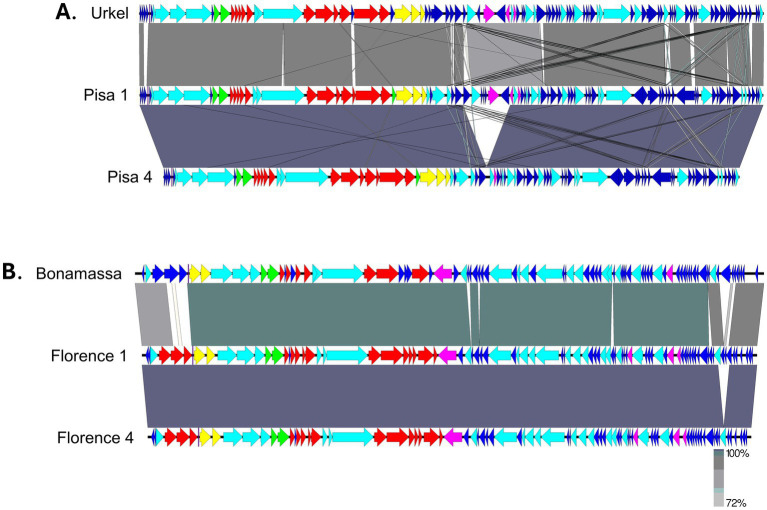
Pisa **(A)** and Florence **(B)** phages genome alignments with their reference phages, Urkel and Bonamassa, respectively. Green: capsid and scaffold proteins; Red: tail proteins; Yellow: lysis-associated proteins; Mauve: lysogeny-associated proteins; Purple: tRNAs; Light blue: other proteins; Blue: hypothetical proteins.

For Pisa1 and Pisa4, 93 and 85 coding sequences (CDSs) were predicted, respectively, along with a single tRNA sequence in each genome. The lower CDS count in Pisa4 is due to its genome being approximately 4.63 Kb shorter than that of Pisa1 (Table 2), resulting in the loss of several CDSs. In particular, one of the missing CDSs encodes an integrase, which is typically part of the lysogeny cassette of mycobacteriophages, including both Pisa1 and the reference phage Urkel. However, Pisa4 maintained other genes from this cassette, including an excisionase and a partial immunity repressor. Notably, the deletion in Pisa4 removed the 3′ end of the gene encoding the immunity repressor, which is intact in Pisa1 (282 nucleotides long). The homologous region in Pisa4 spans only 246 nucleotides, lacking 36 nucleotides that include the stop codon. In addition, a G at position 233 and the absence of a T between nucleotides 239 and 240 distinguish the Pisa1 sequence, potentially altering the amino acid composition without disrupting the open reading frame. The loss of the canonical stop codon in Pisa4 extends the sequence to the first in-frame stop codon 387 bp downstream, resulting in a putative coding sequence of 633 bp. This would predict a much longer protein which, however, may still retain functionality. Since integrase and immunity repressor genes are involved in lysogeny, the ability of Pisa4 to undergo a lysogenic cycle may be affected. Additionally, Pisa phages encoded a holin and two endolysins (A and B), which are essential for host cell lysis. Regarding Florence phages, Florence1 was predicted to have 88 CDSs and one tRNA, while Florence4 was nearly identical to Florence1, except for the absence of three CDSs, all annotated as hypothetical proteins. Both lacked an excisionase gene which is found instead in the reference phage Bonamassa. Finally, endolysins A and B were identified in their genomes, while no holin was predicted. Importantly, no virulence factor or antibiotic resistance genes were detected in any of the phage genomes.

### 3.2 Pisa phages exhibit a siphoviral morphology

To determine the phage morphology, transmission electron microscopy was employed. Pisa phages showed siphoviral morphotypes characterized by icosahedral heads connected to long, non-contractile tails. Specifically, Pisa1 exhibited a capsid with an average diameter of 72.7 nm and a tail length of approximately 200.8 nm, while Pisa4 displayed a capsid diameter of 68.9 nm and a tail measuring around 214.3 nm (Figure 2).

**Figure 2:**
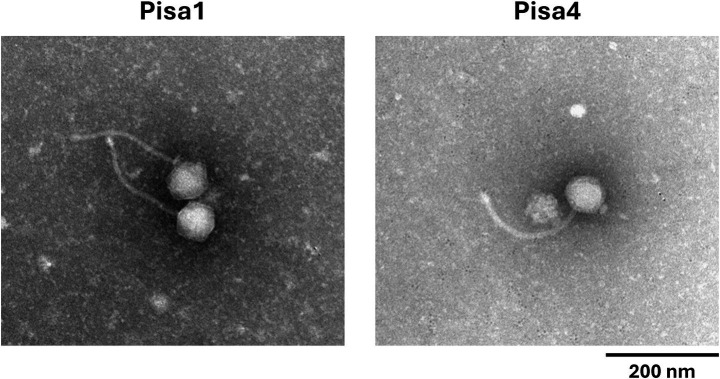
Transmission electron micrographs of Pisa1 and Pisa4 phages.

### 3.3 Phages resist a wide range of pH values and temperatures

To determine whether pH or temperature affected infectivity, these phages were challenged with different conditions and titrated using spot assays. As shown in Figure 3A, the viability of Pisa phages remained stable between pH 4 to 12 but was completely lost in the extremely acidic conditions of pH 3. On the other hand, Florence1 and Florence4 showed partial inactivation at pH 4 (1.66 and 1.55 log_10_ reductions, respectively), pH 5 (0.96 and 1.14 log_10_ reductions) and pH 12 (2.26 and 2 log_10_ reductions). Temperature stability varied among the phages following incubation at different temperatures (Figure 3B). Specifically, Pisa phages remained stable from 4 °C to 45 °C, while they were completely inactivated at higher temperatures. In contrast, Florence phages exhibited greater thermal tolerance. Florence1 showed partial inactivation at 45 °C (2.16 log_10_ reduction) and 55 °C (5.31 log_10_ reduction), with complete loss of viability at 65 °C. Florence4, instead, displayed partial inactivation at 55 °C and 65 °C (3.07 and 3.57 log_10_ reductions, respectively).

**Figure 3:**
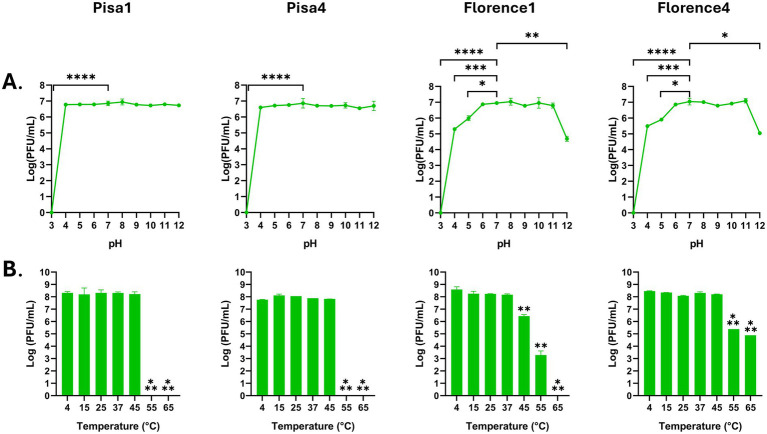
Phage stability at different pH values **(A)** and temperatures **(B)**. The assays were performed in three experimental replicates, each one in duplicate. Statistically significant differences are indicated (Student’s *t* test; *p < 0.05; **p < 0.005; ***p < 0.001; ****p < 0.0001).

### 3.4 Pisa and Florence phages exhibit distinct phage cycle profiles

To investigate the replicative cycle of the four phages in their bacterial host, one-step growth curves were performed (Figure 4). Specifically, Pisa phages required approximately 30 min to replicate their genome within the host cell, synthesize their proteins and assemble new virions, which were subsequently released upon bacterial lysis. In contrast, Florence phages had a shorter latent period of less than 30 min. Following bacterial lysis, the PFU/mL increased and reached a plateau after around 1.5 h for both Pisa and Florence phages, stabilizing at about 1 × 10^7^ and 1 × 10^6^ PFU/mL, respectively. In addition, the burst size, defined as the number of newly produced virions per infected cell, was 151 virions per cell for Pisa1, 125 for Pisa4, 109 for Florence1 and 49 for Florence4.

**Figure 4:**
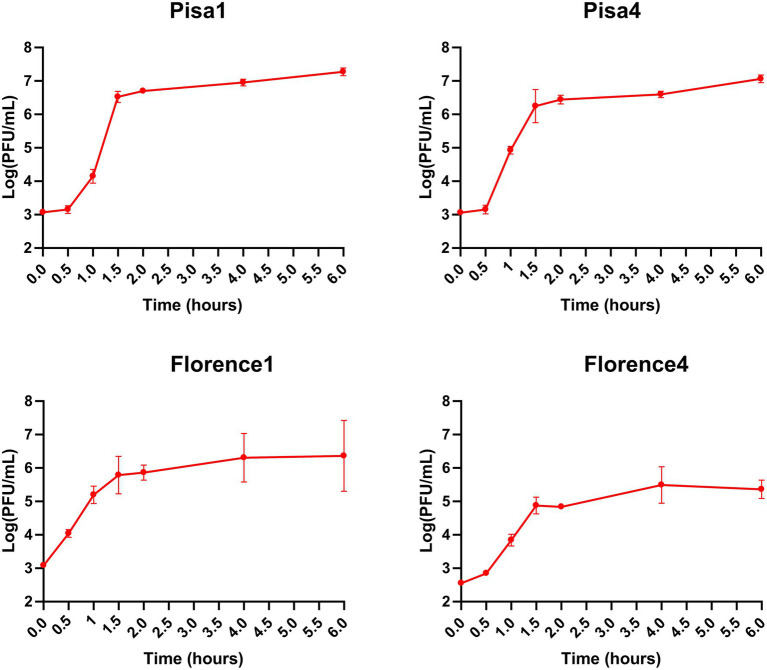
One-step growth curve of the four phages using the host strain *M. smegmatis* mc^2^155. The assay was performed in three experimental replicates, each one in duplicate.

### 3.5 Pisa4 reduces host viability in planktonic conditions

Given the absence of the integrase in the genome of Pisa4 and the presence of a partial immunity repressor, Pisa4 was considered potentially relevant for phage therapy, and was selected for further characterization. First, the lytic activity versus its host in planktonic conditions was performed. As shown in Figure 5, untreated bacteria (growth control) load increased from approximately 1 × 10^6^ CFU/mL to about 1 × 10^8^ CFU/mL within 24 h. In contrast, bacteria samples treated with all the tested phage titres exhibited a reduction of 1 log_10_ CFU/mL and 3 log_10_ CFU/mL in bacterial viability in comparison to the initial inoculum and to the growth control, respectively. This reduction was maintained throughout the experiment, suggesting the ability of Pisa4 to control bacterial growth for up to 48 h. The most prominent effect was observed with the highest MOI, resulting in a rapid decrease in CFU/mL within the first 3 h (1.46 log_10_ reduction), stabilizing at 1 × 10^5^ CFU/mL after 6 h.

**Figure 5:**
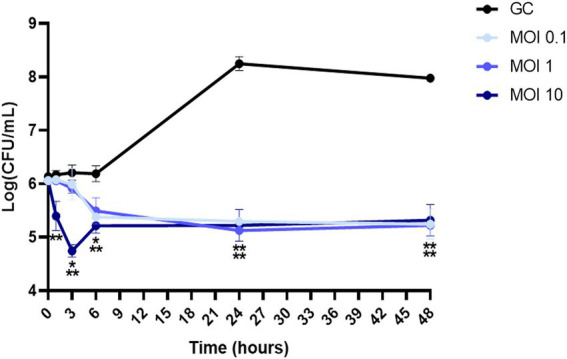
Growth kinetics of *M. smegmatis* mc^2^155 treated with Pisa4. The assay was performed in three experimental replicates, each one in duplicate. GC: growth control. Statistically significant differences are indicated (Multiple Student’s *t* test; **p < 0.005; ***p < 0.0005; ****p < 0.0001).

### 3.6 Pisa4 enters THP-1 macrophages and co-localizes intracellularly with its host with differential efficacy depending on co-incubation or post-infection administration protocols

To determine whether Pisa4 could be endocytosed by macrophages, it was labeled with SYBR Gold, incubated with THP-1 cells for 3 h and analyzed by confocal microscopy. As shown in Supplementary Figure S1, phage uptake was observed in 100% of the cells, confirming efficient internalization by human macrophages. In order to evaluate the ability of Pisa 4 to replicate within macrophages, fluorescently stained phages and *M. smegmatis* strain mc^2^155 expressing the DsRed fluorescent protein were either co-incubated with THP-1 cells or added following bacterial infection. Confocal analysis was performed 3 h after phage administration. In the co-incubation experiment, 18.8% of THP-1 cells were positive for the presence of bacteria, while 100% of the cells internalized phages. Among the bacteria-positive cells, 83.7% showed intracellular co-localization of bacteria with phages, as shown in Figure 6A. On the other hand, in the post-infection treatment, phage uptake was still at 100%, 8.1% of the cells were positive for bacteria, but only 31.6% of these cells exhibited bacteria/phage co-localization, as shown in Figure 6B. Overall, co-localization was significantly higher in the co-incubation condition compared to the post-infection treatment (medians 100% vs. 33.3%; Mann–Whitney U test, *p* = 0.0021).

**Figure 6:**
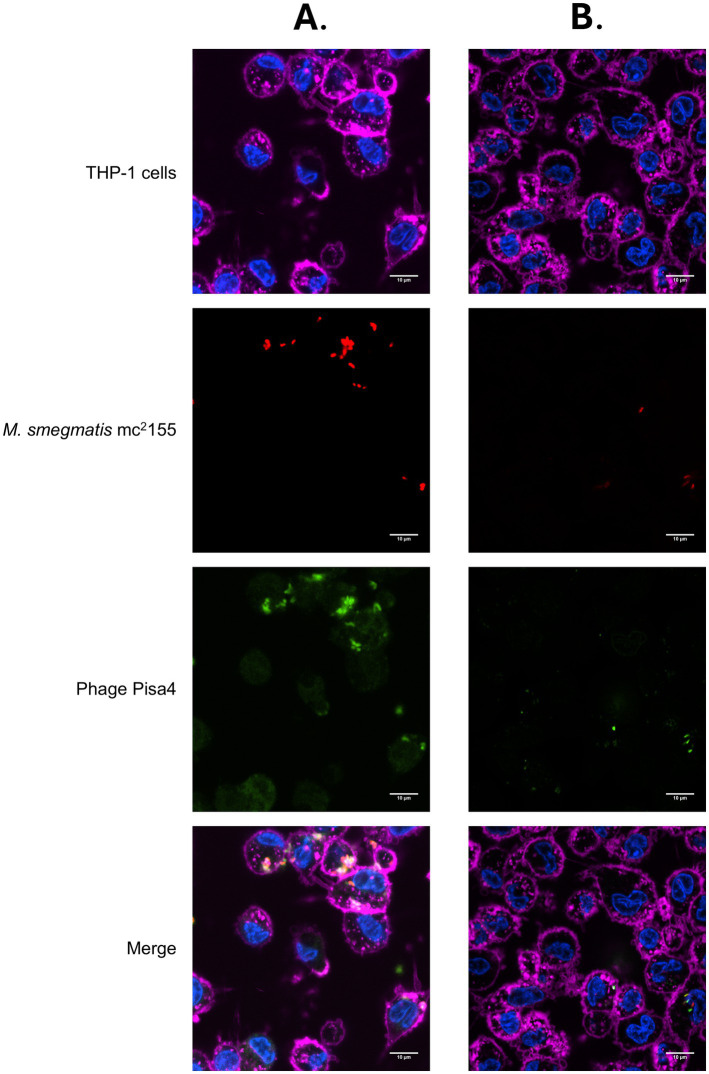
Representative images showing intracellular co-localization of SYBR Gold-stained phages (green) with DsRed-expressing *M. smegmatis* mc^2^155 (red) in THP-1 macrophages during co-incubation **(A)** and post-infection treatment **(B)** experiments. Human cells were stained with Hoechst 33342 (blue) and CellMask Deep Red (magenta). Merged images show areas of phage-bacterium co-localization as white or yellow signals. The scale bar corresponds to 10 μm. This assay was performed in two experimental replicates, each one in duplicate.

### 3.7 Pisa4 replicates intracellularly in THP-1 macrophages when co- or pre-incubated with its host

To further investigate intracellular phage-bacteria interactions, phage replication was assessed under the same infection conditions used for confocal microscopy (co-incubation and post-infection administration), with the addition of a pre-incubation setting. In this additional condition, phages and bacteria were allowed to interact prior to exposure to THP-1 cells, enabling phage-bacteria contact before host cell infection. In these experiments, human cells were lysed at different time points to enumerate intracellular PFU/mL and CFU/mL, and extracellular PFU/mL in the culture medium were also determined.

In the left panels of Figure 7, intracellular phage titers over time are shown (green columns). In both co-incubation (A) and pre-incubation (B) experiments, a statistically significant increase in phage titer was consistently observed in phage-treated bacteria compared to phage administration alone. The increase ranged from 2.3 to 2.6 log_10_ in the co-incubation assay and from 2.1 to 3.5 log_10_ in the pre-incubation assay. Over the course of the experiment, the intracellular phage titers in phage-treated bacteria slightly decreased. On the other hand, in the post-infection treatment experiment (C), no difference in intracellular PFU/mL was observed between phage-treated bacteria and phage administration alone. Regarding extracellular phage titers, they were evaluated over time in both co-incubation (A, left panel) and pre-incubation (B, left panel) experiments (blue columns). At time 0, corresponding to the end of the 3-h incubation of bacteria/phages with human cells, culture media were collected and PFU/mL were determined. At this point, only a slight, non-statistically significant increase in phage titers was observed in phage-treated bacteria compared to phage administration alone. The wells corresponding to the later time points were instead washed and treated with antibiotics to eliminate extracellular bacteria. In the supernatants of phage-treated samples, statistically significant increases in PFU/mL were consistently observed, indicating the release of phages by human cells. Specifically, titers were 3.4–4.4 log_10_ higher in the co-incubation assay and 3.6–4.3 log_10_ in the pre-incubation assay, compared to controls. As with intracellular phage titers, extracellular phage levels associated with phage-treated bacteria also decreased slightly over time. Similarly, no differences in extracellular PFU/mL were observed in the post-infection treatment experiment, consistent with the intracellular titer results (C, left panel, blue columns). Note that, unlike the co-incubation and pre-incubation settings, in the post-infection treatment assay the phage-containing medium was added following antibiotic treatment and maintained throughout the experiment, which explains the persistence of extracellular phages.

**Figure 7:**
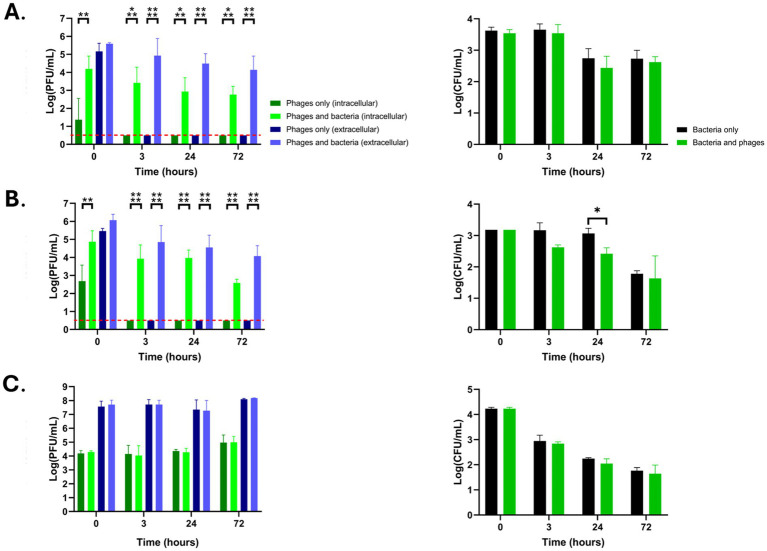
Intracellular/extracellular phage titers (left panels) and intracellular viability of *M. smegmatis* mc^2^155 (right panels) over time for co-incubation **(A)**, pre-incubation **(B)** and post-infection treatment **(C)** experiments. These assays were performed in three experimental replicates, each one in duplicate. Color legends are shown on the right side of the top graphics. Statistically significant differences are indicated [intracellular and extracellular phage titers were analyzed using ordinary One-way ANOVA test (**p < 0.005; ***p < 0.0005; ****p < 0.0001), while intracellular bacterial viability was analyzed using the Multiple Student’s *t* test (*p < 0.05)].

In the right panels of Figure 7, the intracellular viability of *M. smegmatis* mc^2^155 over time is shown for co-incubation (A), pre-incubation (B) and post-infection treatment (C) experiments. A slight yet statistically significant reduction (0.6 log_10_) was observed only at 24 h when bacteria were pre-incubated with phages prior to their addition to human cells (B). Notably, in the post-infection treatment (C), a reduction in bacterial viability was observed in the growth control 3 h after phage administration, an effect that was not evident in the other two conditions (A and B). This reflects the distinct experimental setups. Indeed, in the post-infection treatment, 3 h after phage administration corresponds to a total of 6 h of intracellular residence, during which bacteria are exposed to macrophage-mediated killing. In contrast, in conditions A and B, 3 h after phage administration corresponds to only 3 h of intracellular infection, resulting in lower macrophage bactericidal activity.

### 3.8 Pisa4 does not induce neutrophil extracellular trap release

To investigate whether Pisa4 can trigger neutrophil extracellular traps (NETs) release, primary human neutrophils from six healthy donors were exposed to 10^3^ phages/cell. PMA was included as positive control. After 4 h of incubation, cells were fixed, stained and scored for DAPI positivity (total cells) and for double positivity to DAPI and anti-myeloperoxidase (MPO, a marker of NETosis) (NET-releasing cells). As shown in Figure 8, Pisa4 treatment did not increase NET release compared to the negative control, unlike PMA treatment.

**Figure 8:**
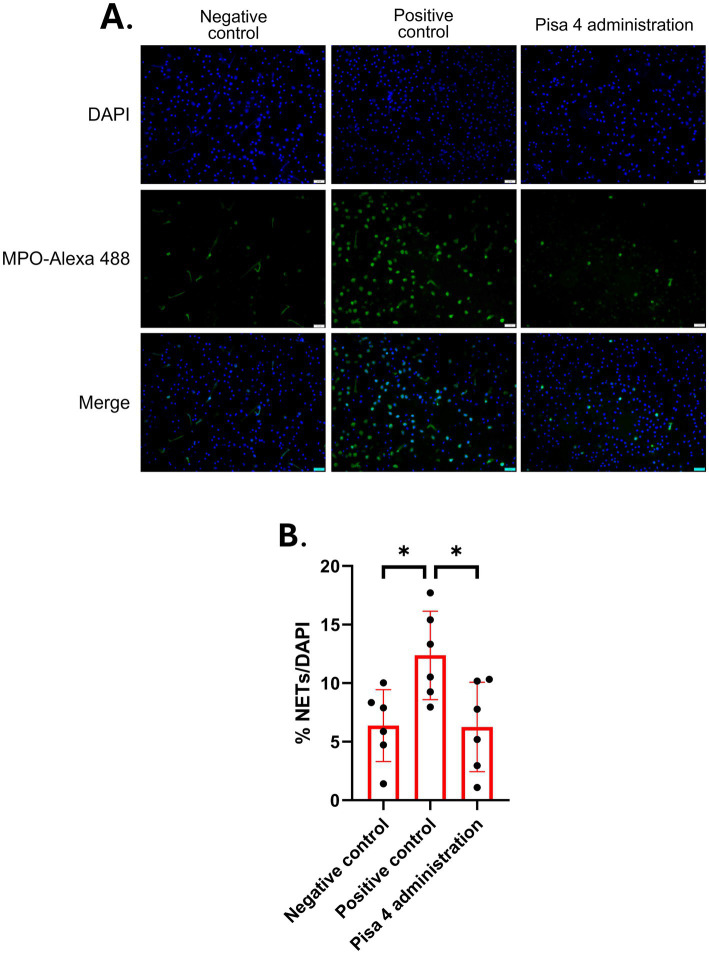
NET release by primary human neutrophils following different treatments. **(A)** Representative images showing total cells and NET-releasing neutrophils. Nuclei were stained with DAPI (blue) and NETs were visualized using an anti-MPO antibody conjugated to Alexa 488 (green). Merged images show double-positive signals in cyan. The scale bar corresponds to 20 μm. **(B)** Percentage of NET-releasing cells after exposure to medium only (C-), 100 nM PMA (C+) or Pisa4 (10^3^ phages/cell). Neutrophils from six healthy donors were tested in duplicate. Statistically significant differences are indicated (One-way ANOVA; *p < 0.05).

### 3.9 Pisa4 does not induce IFN-*γ* and TNF-*α* release, but modulates IL-6 production

To evaluate whether Pisa4 stimulates cytokine production, primary human peripheral blood mononuclear cells (PBMCs) from six healthy donors were exposed to three phage concentrations (10^1^, 10^2^ and 10^3^ PFUs/cell). Two negative controls (cells only and phage buffer) and one positive control (*M. smegmatis* mc^2^155 at MOI 0.1) were included. Cytokine levels (IFN-γ, TNF-α and IL-6) were quantified by ELISA after 24 and 48 h. At 48 h, Pisa4 treatment did not induce IFN-γ release, with concentrations comparable to the negative controls (0–29.4 pg/mL vs. 17.2–19.9 pg/mL) and significantly lower than the positive control (116.9 pg/mL) (Figure 9A). Similarly, TNF-α production remained low and not statistically different from negative controls (124.3–818.2 pg/mL vs. 50.2–69.7 pg/mL), in contrast with the strong induction observed with the positive control (2212.6 pg/mL) (Figure 9B). At 24 h, IFN-γ levels showed no statistically significant differences across conditions, while TNF-α followed a pattern similar to that observed at 48 h (data not shown). On the other hand, IL-6 production was affected by Pisa4. Indeed, phage exposure resulted in a dose-dependent increase in IL-6 (27.9, 792.9 and 1409.9 pg./mL, respectively), reaching levels comparable to the positive control (937.4 pg./mL), while the negative controls remained consistently low (69.2 and 67.6 ng/mL) (Figure 9C). IL-6 concentrations measured at 24 h were comparable to those at 48 h (data not shown).

**Figure 9:**
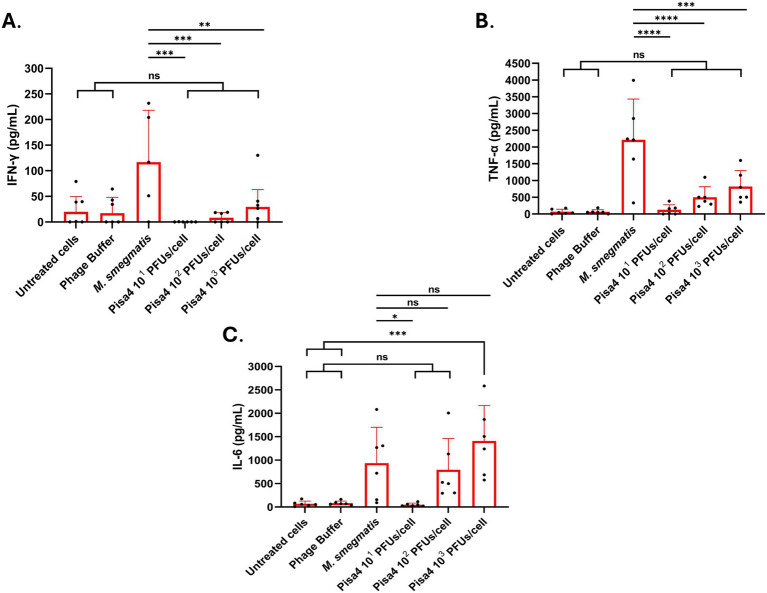
IFN-*γ* **(A)**, TNF-*α* **(B)** and IL-6 **(C)** production by primary human peripheral blood mononuclear cells (PBMCs) following different treatments, including medium only (negative control), phage buffer (negative control), *M. smegmatis* mc^2^155 at MOI 0.1 (positive control) or three increasing concentrations of Pisa4 (10^1^–10^3^ PFUs/cell). PBMCs from six healthy donors were tested in triplicate. Statistically significant differences are indicated (Two-way ANOVA; ns, not significant; *p < 0.05; **p < 0.01; ***p < 0.001; ****p < 0.0001).

### 3.10 Phages showed a narrow host range versus *M. abscessus* strains

To evaluate phage activity against pathogenic mycobacteria, Pisa4 and the other three phages were tested against *M. abscessus* ATCC19977 (S strain) and 47 *M. abscessus* clinical isolates, revealing a narrow host range (Figure 10). Pisa1 was able to infect five of the tested *M. abscessus* strains, including four S strains (2871, 2953, 3208S and R2S) and one R strain (3208R), with EOP values ranging from 0 to 0.1. Pisa4 exhibited infectivity toward two S strains (3208S and R2S) and one R strain (3208R); EOP values similarly ranged between 0 and 0.1, except for strain 3208R, for which the EOP was between 0.1 and 1. In contrast, Florence phages showed no activity against any of the tested strains.

**Figure 10:**
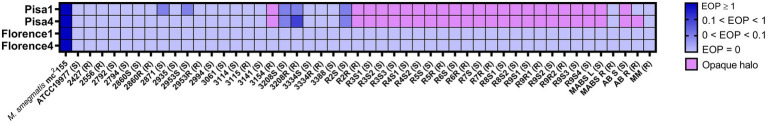
Efficiency of plating (EOP) assays of phages against 48 strains of *M. abscessus*. This assay was performed in two experimental replicates, each one in duplicate. At the bottom of the image, strains IDs are listed alongside their colony morphotype (R, rough; S, smooth). The color legend on the right represents EOP values or the presence of an opaque halo.

Moreover, Pisa phages produced opaque halos on 25 strains, without visible plaques at the lower dilutions. To confirm phage infectivity, liquid infections assay (MOI 0.1) were performed (Table 3). Pisa1 replicated in seven isolates, including six S strains (R3S1, R3S2, R3S3, R4S1, R4S2 and R9S3) and one R strain (R2R). Pisa4, on the other hand, propagated in five isolates, including four S strains (R3S1, R3S2, R4S1 and R9S3) and one R strain (R2R). Altogether, these results increased the total number of clinical isolates susceptible to Pisa phages to twelve.

**Table 3:** Log₁₀ increase in phage titers at 24 h post-infection against *M. abscessus* clinical isolates.

*M. abscessus* strain	Pisa1	Pisa4
R2R	2.3	2.2
R3S1	0.5	1.7
R3S2	1.2	1.3
R3S3	0.9	0
R4S1	1.6	2.1
R4S2	1.1	0
R9S3	1.0	1.8

## 4 Discussion

In this study, four phages (Pisa1, Pisa4, Florence1 and Florence4) were isolated from soil samples using *M. smegmatis* mc^2^155 as host, a rapidly growing, non-pathogenic and prophage-free bacterium, selected for its genetic and physiological similarities to pathogenic mycobacteria (Sparks et al., 2023) and for its ability to produce clean phage lysates without the risk of prophage contaminations (Hatfull, 2023). In addition, several *M. abscessus* strains, including both smooth (S) and rough (R) morphotypes, were used for soil enrichment, but no phages were recovered. S strains are generally less susceptible to phage infection due to presence of surface glycopeptidolipids, which may limit access to the phage receptors (Dedrick et al., 2021, 2023b); therefore, the difficulty in isolating phages using S strains was not unexpected. However, the failure to recover phages even with R strains suggests that environmental phages capable of infecting *M. abscessus* are rare and/or display a highly restricted host range toward this species. This finding is consistent with the limited literature on phage isolation using *M. abscessus* as a host. To date, only one environmental phage (P3MA) has been isolated and characterized using a clinical *M. abscessus* strain (Broncano-Lavado et al., 2025).

Genomic analysis revealed that the described phages possess a dsDNA genome and do not carry known virulence factors or antibiotic-resistance genes. All four encode elements of a lysogeny cassette. This cassette is complete only in Pisa1, which includes the integrase, excisionase and immunity repressor genes. In contrast, both Florence1 and Florence4 lack the excisionase gene, while Pisa4 lacks the integrase gene. Among these, the absence of the integrase and the presence of a partial immunity repressor gene in Pisa4 is particularly noteworthy, suggesting a possible impaired ability to establish lysogeny. Additionally, taxonomic analysis indicated that Pisa and Florence phages represent new species within the *Anayavirus* and *Benedictvirus* genera, respectively. Pisa phages belong to cluster K1, while Florence phages belong to cluster A5. Notably, phages belonging to the cluster K have been reported to infect and lyse *M. tuberculosis* (Jacobs-Sera et al., 2012; Guerrero-Bustamante et al., 2021), suggesting that Pisa phages may be promising candidates for further *in vitro* testing against this pathogen.

Pisa and Florence demonstrated rapid bacterial killing together with substantial phage progeny production, two essential parameters for clinical applications, and they are consistent with previous observations reported in the literature (Bonacorsi et al., 2024). Considering that phagosomes infected with mycobacteria typically maintain an acidic environment of pH 4–6 (Vandal et al., 2009), these findings suggest that the phages could retain their infectivity within the endocytic compartment. These results align with previous studies, although some mycobacteriophages described in the literature are already inactivated at pH 5 (Bonacorsi et al., 2024). Finally, the thermal stability assay showed that Pisa phages remained viable between 4 °C and 45 °C, while Florence phages exhibited greater heat tolerance and were just partly inactivated at higher temperatures, like in the case of Florence4 at 65 °C. These results are also consistent with what has been reported for other mycobacteriophages (Bonacorsi et al., 2024).

Given the absence of the integrase and the presence of only a partial immunity repressor in the genome of Pisa4, suggesting potentially impaired lysogenic capabilities, this phage was further characterized. Initially, its bactericidal activity was evaluated under planktonic conditions, where the phage showed a marked reduction in *M. smegmatis* mc^2^155 viability. Subsequently, Pisa4 was purified by CsCl gradient centrifugation to remove cellular debris and its interaction with THP-1 macrophages was investigated using confocal microscopy. First, the ability of Pisa4 to enter human cells was confirmed. This was expected, as phages are known to be internalized primarily through non-specific macropinocytosis (Miernikiewicz and Dąbrowska, 2022). Then, THP-1 cells were infected with *M. smegmatis* mc^2^155 to evaluate the potential intracellular co-localization of phages and bacteria. Even though this bacterium is non-pathogenic and does not reflect clinically relevant mycobacteria such as *M. abscessus* and *M. tuberculosis*, it provides a useful model to investigate preliminary phage-bacteria dynamics within human cells. To this aim, different experimental setups were designed. In particular, the bacteria/phage co-incubation experiment simulates a scenario where both bacteria and phages are present extracellularly at the site of infection, allowing to assess whether internalized phages co-localize with bacteria within intracellular compartments. In the post-infection treatment, instead, phages were administered after bacterial infection and killing of the extracellular ones to evaluate whether phages could be internalized by human cells and reach intracellular bacteria without any extracellular stimulus or carrier. Co-localization represents a necessary, although not sufficient, condition for subsequent bacterial infection and lysis. As a result, Pisa4 co-localized with its host within THP-1 cells in the co-incubation condition (18.8% of the cells were bacteria-positive, of which 83.7% showed co-localization with phages). Interestingly, confocal microscopy also revealed phage-bacteria co-localization in the post-infection treatment, although to a lower extent compared to the co-incubation condition (31.6% of 8.1% bacteria-positive cells). In the co-incubation assay, simultaneous uptake of bacteria and phages likely facilitates their co-localization within the same intracellular compartments. In contrast, during the post-infection assay, where cells are first infected with bacteria and subsequently exposed to phages, the likelihood of converging endocytic pathway is reduced. Co-localization events of mycobacteriophages and mycobacteria have been previously described using mCherry reporter phages and GFP-expressing *M. abscessus via* confocal microscopy, as well as through transmission electron microscopy (Schmalstig et al., 2024). In this study, SYBR Gold-labelled phages and DsRed-expressing bacteria were used for confocal imaging, offering further evidence of phage/bacterium co-localization. Compared to mCherry reporter system, which marks bacteria that are infected and expressing the reporter gene, SYBR Gold staining enables direct visualization of phage-associated nucleic acids, allowing detection of phage-bacterium interactions even in the absence of productive infection. Although SYBR Gold does not discriminate between intact phage particles and nucleic acids derived from degraded phages, this approach still provides spatial information on phage localization within infected cells.

Following confirmation of phage entry into human cells and intracellular co-localization with bacteria, it was investigated whether Pisa4 could complete a productive infection cycle. When assessing the intracellular phage load, statistically significant increases were always observed in both co-incubation and pre-incubation assays (2–3.5 log_10_), indicating effective phage replication. Although cells were washed three times with PBS to remove extracellular phages prior to downstream processing, it cannot be completely excluded that a small fraction of phage particles remained; however, this residual contribution is expected to be minimal. In addition, the increase in intracellular phage load could be attributed not only to intracellular phage activity, but also to extracellular phage replication before extracellular bacteria were eliminated. Indeed, since bacteria and phages were incubated with human cells for at least 3 h before intracellular PFU/mL enumeration, extracellular phages may have replicated, leading to subsequent phage production and internalization by human cells. This occurred even though no difference in CFU/mL was observed between phage-treated and untreated bacteria at time 0. In this context, it is important to consider that for each lysed bacterium, approximately 125 PFUs are released, meaning that even a minor reduction in CFUs can result in a significant PFU increase. Over the course of the experiment, the intracellular phage titers in phage-treated bacteria decreased, possibly due to phage release by human cells or phage inactivation (Fajardo-Lubian and Venturini, 2023). Regarding the post-infection treatment assay, instead, the absence of intracellular phage titer increase indicates that the phage could not complete the replicative cycle. This may be due to the limited ability of phages to efficiently reach intracellular bacteria. Indeed, confocal microscopy revealed that only 31.6% of bacteria-positive cells showed phage co-localization, corresponding to just 2.6% of the total THP-1 cell population, compared with 15.7% observed in the co-incubation setting.

When investigating the extracellular phage titers, the absence of phage titer increases at time 0 suggests that the statistically significant increase in intracellular phage titers, at the same time, is primarily due to intracellular phage activity. This applies to both co-incubation and pre-incubation experiments. At later time points, statistically significant increases in PFU/mL (3.5–4.5 log_10_) were always observed compared to the control, indicating that intracellular phages were released by human cells. It cannot be excluded that infected human cells potentially release bacteria in the extracellular environment after cell lysis, and these might be later infected by the phage. However, given that in the post-infection treatment assay no increase in extracellular phage load was noticed, it is more likely that phages derive intracellularly. Over time, the extracellular phage titers associated with phage-treated bacteria decreased, potentially due to phage inactivation.

Finally, when evaluating the intracellular bacterial load, a slight but statistically significant reduction of 0.6 log_10_ was observed only at 24 h when bacteria were pre-incubated with phages prior to their addition to human cells. Overall, bacterial killing was limited, which might be partly due to the putative temperate nature of the phage, as well as to partial phage inactivation within the hostile intracellular environment. Indeed, although internalized phages can transit through various subcellular compartments and may be exocytosed, many are likely inactivated within lysosomes (Fajardo-Lubian and Venturini, 2023). Additionally, differently from *M. abscessus* and *M. tuberculosis*, *M. smegmatis* does not replicate efficiently within THP-1 macrophages (Sousa et al., 2019; Feng et al., 2020), which is consistent with its avirulent nature. In fact, reduced growth and changes in metabolic activity may further impair phage infection (Fajardo-Lubian and Venturini, 2023), ultimately limiting phage bactericidal effects. This aspect could be further clarified by performing functional analyses of the metabolic status of intracellular bacteria. The observed statistically significant reduction in intracellular CFU/mL during the pre-incubation assay may be explained by the fact that the phage had infected the bacterial cells prior to their internalization, being protected from inactivation within the host cell environment, and may have been able to complete its replicative cycle before substantial changes in the bacteria occurred.

Overall, these findings demonstrate that Pisa4 is capable of entering human macrophages, co-localizing with intracellular mycobacteria and, under specific conditions, completing its replicative cycle within the intracellular environment. Therefore, the next step will be to evaluate the therapeutic potential of Pisa4 in intracellular infection models involving clinically relevant pathogens such as *M. abscessus* and *M. tuberculosis*. Schmalstig and colleagues followed a post-infection treatment approach similar to the one used in this work and demonstrated that phage efficacy against intracellular *M. abscessus* GD82, evaluated by bacterial plating, can vary depending on the MOI (10^3^ and 10^5^) and the cell type (THP-1, A549 and murine bone marrow-derived macrophages) (Schmalstig et al., 2024). In general, higher MOIs were associated with greater intracellular bactericidal activity, whereas lower MOIs sometimes failed to impact bacterial viability. Notably, only one phage (ZoeJΔ45) exhibited activity across all three cell types, while another (Muddy) showed no activity in any of them (Schmalstig et al., 2024). It is therefore possible that the concentration of Pisa4 used in this study was insufficient to significantly kill intracellular bacteria, or that this phage is partly inactivated within the specific THP-1 intracellular niche (Fajardo-Lubian and Venturini, 2023). In addition to this work (Schmalstig et al., 2024), where free phages were administered, other investigations have used a “Trojan horse” strategy. Specifically, TM4-infected *M. smegmatis* mc^2^155 was used to deliver the phage intracellularly, leading to a reduction in the viability of both *M. avium* and *M. tuberculosis* within RAW 264.7 mouse macrophages (Broxmeyer et al., 2002). This effect was also confirmed *in vivo* in a mouse model of *M. avium* infection (Danelishvili et al., 2006). In contrast, this work provides a systematic comparison of three delivery approaches and, beyond assessing intracellular bacterial viability, it also quantifies both intracellular and extracellular phage loads at several time points, an aspect that should be evaluated in future studies, allowing to demonstrate phage persistence and replication within human cells.

Neutrophils are early responders at infection sites, where they phagocytose and release neutrophil extracellular traps (NETs), which are chromatin structure coated with antimicrobial proteins that trap and neutralize invading microorganisms (Borkute et al., 2021). They are frequently observed in mycobacterial lesions, although they are generally not effective at fully controlling the infection (Parker et al., 2021). In this work, exposure of primary human neutrophils to a high concentration of purified Pisa4 (10^3^ phages/cell) did not induce NET formation, suggesting that this phage is unlikely to trigger NETosis and thereby promote neutrophil-driven inflammation and tissue damage. This aspect is particularly relevant in pathological conditions characterized by pre-existing hyperinflammation, such as CF, where excessive NET accumulation already contributes to airway obstruction and disease progression (Marcos et al., 2015). CF is mentioned also because *M. abscessus* represents a significant infectious threat in these people, where chronic airway disease favors its persistence and clinical severity (Degiacomi et al., 2019). To the best of our knowledge, only one other study has investigated NET release in response to phage exposure, reporting that increasing concentration of the anti-pseudomonal phage PAK_P1 (up to 10^4^ phages/cell) did not increase in SYTOX Green fluorescence measured by a microplate reader (Roach et al., 2023). Importantly, our study confirms these findings using a complementary approach based on immunofluorescence imaging.

Cytokines such as IFN-*γ*, TNF-*α* and IL-6 play a central role in modulating the immune response (Shamaei and Mirsaeidi, 2021; Vu et al., 2024). Specifically, IFN-*γ* is a critical cytokine for the activation of macrophages and the promotion of Th1 immune responses, enhancing the clearance of intracellular pathogens. TNF-α plays a central role in initiating and sustaining inflammatory responses, contributing to cell recruitment, apoptosis or tissue damage. IL-6 is a cytokine that regulates acute-phase responses, supports B cell differentiation and modulates both pro- and anti-inflammatory pathways (Shamaei and Mirsaeidi, 2021; Vu et al., 2024). In this study, exposure of PBMCs to increasing concentrations of purified Pisa4 (10^1^–10^3^ phages/cell) did not induce IFN-*γ* or TNF-α release, but significantly increased IL-6 production at the highest concentration. However, IL-6 induction may also reflect co-purified host components. Indeed, cesium chloride gradient purification of mycobacteriophages can result in the co-purification of residual bacterial contaminants, possibly including host-derived macromolecules that may retain immunostimulatory activity (Viland et al., 2025). Given that mycobacterial cell envelope components, such as lipoglycans and glycolipids, are known inducers of cytokine production, including IL-6 (Barnes et al., 1992; Zhang et al., 1994; Geisel et al., 2005), it is plausible that the observed increase reflects a purity-related artifact rather than an intrinsic immunomodulatory property of the phage (Barnes et al., 1992; Zhang et al., 1994; Geisel et al., 2005; Viland et al., 2025). Overall, these findings indicate that Pisa4 does not trigger a strong pro-inflammatory response, although this interpretation is based on the analysis of only three cytokines. Since many other mediators contribute to shaping immune activation, further studies are required to define the immunomodulatory profile of Pisa4. IFN-*γ*, TNF-*α* and IL-6 have been described to play a protective role against mycobacterial infections, activating macrophages and promoting the formation of granulomas to contain the bacteria (Cooper et al., 2011; Gidon et al., 2021). In this context, the observation that Pisa4 does not induce the release of all of them could then be considered a limitation for direct pathogen clearance. It should be noted that the infecting bacteria themselves strongly stimulate the immune system, therefore the lack of robust cytokine induction by Pisa4 may be beneficial in this context. Importantly, this profile may be advantageous for phage therapy in chronic conditions characterized by hyperinflammation, as it limits the risk of exacerbations. For instance, in CF, the absence of TNF-α induction after phage administration could be particularly beneficial, given that this pro-inflammatory cytokine is consistently elevated in the lungs of affected people and is closely linked to airway inflammation (Courtney et al., 2004). Conversely, although Pisa4 does not increase IFN-γ levels, its induction could have been advantageous. For example, stimulation of human CF macrophages with IFN-γ, which are normally defective in IFN-γ production post-infection, resulted in a significant reduction of intracellular *Burkholderia cenocepacia* burden, comparable to that observed in non-CF macrophages (Assani et al., 2014). IL-6 expression is also elevated in people with CF (Courtney et al., 2004), so its induction by Pisa4 could potentially have detrimental effects. However, a beneficial role cannot be excluded, given the dual pro- and anti-inflammatory nature of this cytokine (Kaur et al., 2020). Our experimental findings are consistent with previous reports. Van Belleghem et al. (2017) suggested that the observed downregulation of IFN-γ expression in PBMCs following exposure to one *Pseudomonas aeruginosa* phage could be linked to the upregulation of the anti-inflammatory cytokine IL-10, as assessed by transcriptomics. At the protein level, Perea et al. demonstrated increased IL-10 production in PBMCs exposed to one *S. aureus* phage and three *Escherichia coli* phages (Perea et al., 2021). Extending these observations, our results confirm that IFN-γ concentrations remain unchanged after Pisa4 treatment, possibly by increased expression of IL-10. For TNF-α, Belleghem et al. reported phage-specific expression patterns in PBMCs, determined by qRT-PCR: one *Staphylococcus aureus* phage and two *P. aeruginosa* phages increased its expression, whereas two other pseudomonal phages downregulated it (Van Belleghem et al., 2017). Consistently, protein-level studies also showed similar variable outcomes. Perea et al. showed increased levels of TNF-α following treatment with the four phages (Perea et al., 2021), whereas Perea et al. (2021) found that TNF-α was downregulated in human U937 macrophages when treated with a filamentous *P. aeruginosa* phage. Collectively, these results underscore the heterogeneous regulation of TNF-α by different phages. Popescu et al. (2022) observed upregulated IL-6 expression, which is consistent with Pisa4–induced IL-6 release at high titers. Overall, these results suggest that phages may have evolved predominantly anti-inflammatory properties, while retaining some pro-inflammatory potential. A dampened immune response could confer an evolutionary advantage to phages, reducing their clearance by the host immune system while simultaneously limiting the elimination of their bacterial hosts, thereby ensuring further opportunities for phage propagation.

Finally, the host range of all the phages was evaluated against a panel of *M. abscessus* clinical isolates collected from three Italian hospitals, spanning rough/smooth morphotypes as well as different subspecies. This fast-growing, non-tuberculous mycobacterium was selected because it has emerged as a major opportunistic pathogen, particularly in individuals with underlying lung disorders, including CF patients, and is notoriously refractory to antibiotic treatment (Johansen et al., 2020). In this context, infections on semi-solid media can sometimes result in the appearance of opaque halos without distinct plaque formation, leading to the false conclusion that the phage is inactive. As previously observed with *Klebsiella pneumoniae* phages, liquid infection assays are critical in such cases to confirm actual phage activity (Fausti et al., 2025). Overall, EOP and liquid infection assays showed that, among 48 tested *M. abscessus* clinical isolates, Pisa1 was active against twelve strains, while Pisa4 infected eight strains. In contrast, Florence phages failed to lyse any of the tested strains. This isolate set provides important insights into phage susceptibility, yet expanding the panel through multi-center sampling and genotyping will be required to capture broader lineage diversity and generalize susceptibility rates. Importantly, EOP values greater than 0.1, such as those observed for Pisa4 on strain 3208R, are generally considered indicative of promising therapeutic potential. For EOP values below 0.1, although phage efficacy might be limited, therapeutic use may still be feasible through the isolation of host range mutants (Jacobs-Sera et al., 2012). Overall, these results highlight the narrow host range of these phages, a common feature among mycobacteriophages. Notably, the mechanisms underlying resistance of *M. abscessus* to phage infection remain poorly characterized and may be related to multiple factors, including the absence of specific phage receptors, mutations affecting receptors or co-receptors (e.g., trehalose polyphleates), as well as the presence of surface-exposed glycopeptidolipids that may hinder phage binding to their targets (Bonacorsi et al., 2024). The limited host range of mycobacteriophages also underscore the need of establishing large mycobacteriophage libraries to achieve broader coverage and expand the number of treatable patients. Importantly, given that phages isolated using *M. smegmatis* mc^2^155 are potentially active against other mycobacterial species, including *M. tuberculosis* and both slow- and fast-growing non-tuberculous mycobacteria (Bonacorsi et al., 2024), the therapeutic potential of these phages may extend beyond *M. abscessus*.

In the context of phage therapy, it is crucial to use strictly lytic phages to avoid the reduced bactericidal activity and potential gene transfer risks associated with prophage induction (Strathdee et al., 2023). Since Pisa4 retains a partial immunity repressor gene, its complete removal would be necessary to ensure a fully lytic profile and strengthen its suitability as a phage therapy candidate. Conversion of this phage into a strictly lytic variant, could be achieved using the bacteriophage recombineering of electroporated DNA (BRED) technique (Marinelli et al., 2008), an approach that has already been successfully applied to remove immunity repressor genes in several mycobacteriophages used in therapeutic contexts for the treatment of mycobacterial infections (Dedrick et al., 2023a).

## Data Availability

The sequencing data analyzed in this study can be found in the NCBI GenBank (https://www.ncbi.nlm.nih.gov/genbank/), under accession numbers PX093955, PX093956, PX093957, PX093958, KX657796.1, and OL455889.1.

## Supplementary material

SUPPLEMENTARY FIGURE S1. Representative micrograph showing the uptake of SYBR Gold-stained phages (green) in THP-1 macrophages. Human cells nuclei were stained with Hoechst 33342 (blue) and membranes with CellMask Deep Red (magenta). The scale bar corresponds to 10 μm. This assay was performed in two experimental replicates, each time in duplicate.
